# Chloroplast genome characterization of *Uncaria guianensis* and *Uncaria tomentosa* and evolutive dynamics of the Cinchonoideae subfamily

**DOI:** 10.1038/s41598-023-34334-1

**Published:** 2023-05-24

**Authors:** Andrezza Arantes Castro, Rhewter Nunes, Larissa Resende Carvalho, Cíntia Pelegrineti Targueta, Ramilla dos Santos Braga-Ferreira, Amanda Alves de Melo-Ximenes, Leonardo Carlos Jeronimo Corvalán, Bianca Waleria Bertoni, Ana Maria Soares Pereira, Mariana Pires de Campos Telles

**Affiliations:** 1 Laboratório de Genética and Biodiversidade (LGBio), Instituto de Ciências Biológicas – Universidade Federal de Goiás (UFG), Goiânia, GO 74045-155 Brazil; 2 Instituto Federal de Goiás – Campus Cidade de Goiás (IFG), Goiás, GO 74600-000 Brazil; 3 Universidade de Ribeirão Preto, UNAERP, Ribeirão Preto, SP 14096-900 Brazil; 4 Escola de Ciências Médicas e da Vida, Pontifícia Universidade Católica de Goiás (PUC - GO), Goiânia, GO 74605-050 Brazil

**Keywords:** Comparative genomics, Genome evolution

## Abstract

*Uncaria* species are used in traditional medicine and are considered of high therapeutic value and economic importance. This work describes the assembly and annotation of the chloroplast genomes of *U. guianensis* and *U. tomentosa*, as well as a comparative analysis. The genomes were sequenced on MiSeq Illumina, assembled with NovoPlasty, and annotated using CHLOROBOX GeSeq. Addictionaly, comparative analysis were performed with six species from NCBI databases and primers were designed in Primer3 for hypervariable regions based on the consensus sequence of 16 species of the Rubiaceae family and validated on an in-silico PCR in OpenPrimeR. The genome size of *U. guianensis and U. tomentosa* was 155,505 bp and 156,390 bp, respectively. Both Species have 131 genes and GC content of 37.50%. The regions *rpl32-ccsA, ycf1,* and *ndhF-ccsA* showed the three highest values of nucleotide diversity within the species of the Rubiaceae family and within the *Uncaria genus*, these regions were *trnH-psbA*, *psbM-trnY,* and *rps16-psbK.* Our results indicates that the primer of the region *ndhA* had an amplification success for all species tested and can be promising for usage in the Rubiaceae family. The phylogenetic analysis recovered a congruent topology to APG IV. The gene content and the chloroplast genome structure of the analyzed species are conserved and most of the genes are under negative selection. We provide the cpDNA of Neotropical *Uncaria* species, an important genomic resource for evolutionary studies of the group.

## Introduction

The Rubiaceae family is divided into three subfamilies: Ixoroideae, Rubioideae and Cinchonoideae^1^. The genus *Uncaria* Screb. belongs to the subfamily Cinchonoideae. This genus’ species are distributed along the tropics, covering three continents, with 29 species found in Asia, three in Africa and two in the Americas, totaling 34 species^2–4^ . *Uncaria* species are woody lianas, bindweed and bushes that need support to grow. Their main character is the pairs of hook-shaped thorns formed by the abortive peduncles^2,5^.

The species of this genus are known for their pharmacological properties and are, consequently, economically important. Up to date, it has been identified more than 200 chemical components that act mainly in the nervous and cardiovascular systems within *Uncaria’*s species. In general, these compounds have antioxidant, anti-inflammatory, anti-diabetics, anti-microbial and immunomodulatory actions being widely used for traditional medicine^6–8^.

In Chinese pharmacopoeia, the species *U. hirsuta* Havil., *U. macrophylla* Wall., *U. sessilifructus* Roxb., *U. sinensis* (Oliv.) Havil. e *U. rhynchophylla* (Miq.) Miq. Ex Havil. are the main herbs that constitute the popular Chinese drug *Gouteng*, which is used in many herbal formulas of Asian traditional medicine^6,7^. Other species of the genus are also negotiated as substitutes due to their similar morphological traits. However, this can lead to herbal adulteration and decrease of product quality^9,10^.

In South America, specifically in the Amazon region where *U. guianensis* (Aubl.) J.F.Gmel. and *U. tomentosa* (Willd. Ex Schult. DC.) are native from, it is difficult for tradespeople, which acquire products from extractive communities, to identify the *Uncaria* species, since they all have the same popular name, “cat’s claw”, and are purchased on their dehydrated form^11,12^. Yet, some indigenous communities recognize *U. tomentosa* as the real “cat’s claw”, while others give this name to *U. guianensis*^13^. It is important to highlight that *U. tomentosa* is the most wanted species known by this name, however, it has a more restricted distribution than *U. guianensis*^14,15^.

The Brazilian Pharmacopoeia and the National Relation of Essential Drugs of Brazil have *U. tomentosa* described under the name “cat’s claw” and they report that it is used for arthritis and osteoarthritis treatment, since it shows anti-inflammatory and immunomodulatory roles^16–18^. The National Program of Medicinal Herbs and Brazilian Herbals aims to guarantee secure access and rational use of medicinal herbs, as well as to stimulate commercial production at a large scale, including at the export level^16^. Since *U. tomentosa* is a Brazilian herbal with its use encouraged and authorized by the government, it is essential to have tools available for helping product certification that guarantee efficacy and security.

The commercialization of medicinal herbs of this genus is usually towards tea consumption, pill supplementation or herbal formulas. Most of the products identify the plant compound as “cat’s claw” and do not specify the species, which leads to lack of information for the consumer^11^. It is known that the secondary metabolites bioactive content differs between species^19,20^ and even between parts of the plant^21^. Thus, the clinical efficacy of herbals can be affected when replacement for other morphologically similar species occurs. This can happen due to difficulty with the correct identification and can lead to lack of consumption security^22^.

The DNA barcoding technique has been employed for the identification of species by analyzing one or more specific sequences of a genome. In animals, the mitochondrial gene cytochrome c oxidase I (COI) is the most used. However, in plants, several typeses of chloroplast markers can be used, both from coding and from intergenic regions^23–25^. This technique can be applied for commercial purposes to prevent drug and tea fraud for the identification of threatened species^10,26–30^, as well as to help evolutionary studies^3,31^.

While using chloroplast genome sequences alignments, it is possible to point out hypervariable regions that can be used on the identification of species^29,32,33^, which can enable evolutionary studies, comparative analysis for better comprehension of genome function and origin^34–37^ or even to be used as a super barcode^38^.

The chloroplast genome (cpDNA) is mainly composed by genes involved in photosynthesis. Within angiosperms, their majority show cpDNA in a circular form with a quadripartite structure, which consists of a Large Single Copy (LSC), a Small Single Copy (SSC) and two Inverted Repeats (IRa/IRb)^39^. On GenBank, an NCBII database, there have been deposited 4650 chloroplast genomes of terrestrial plants. For the *Uncaria* genus, up to date, there are only five species with complete cpDNA deposited on NCBI, *U. rhynchophylla* 40, *U. hirsuta*, *U. machrophylla*, *U. scandens* and *U. sessilifructus* all occurring in Asian regions and so far no species occurring in America had their genomes deposited^40,41^*.*

Due to the medical importance related to *Uncaria* species, in this work, we sequenced, assembled, and characterized the chloroplast genomes of the two *Uncaria* species native to the Amazon region, *U. guianensis* and *U. tomentosa.* We also performed a comparative analysis among species of the subfamily Cinchonoideae and rebuilt a phylogenetic tree of the Gentianales order to help provide more genomic data and to understand the evolutionary history of this subfamily. In addition, *primers* were designed for the hypervariable regions of the genomes within this genus to be used forn the identification of species and to help herbal quality control.

## Results

### *U. guianensis* and *U. tomentosa* chloroplast genome features

**Figure 1:**
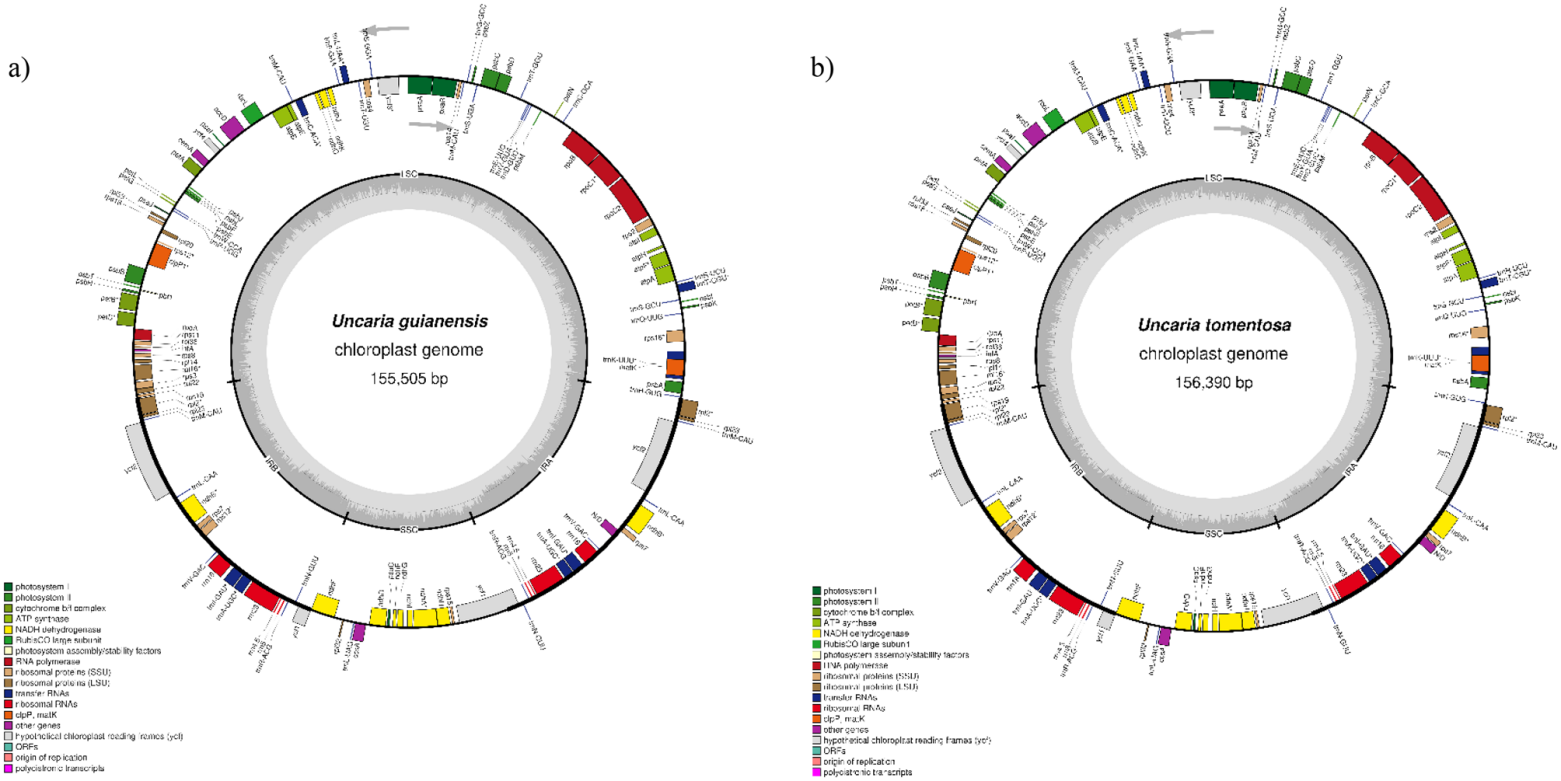
Organellar map of the chloroplast genomes of *U. guianensis* (**a**) and *U. tomentosa* (**b**). LSC, large single copy; SSC, small single copy; IRA and IRB, inverted repeat. The colors indicate genes from different functional groups, as shown in the subtitles. The genes inside the circle are transcribed clockwise, and the genes outside the circle counterclockwise, as indicated by the gray arrows. The inner dark gray circle represents the GC content and the light gray circle the AT content. *Genes that contain introns.

The circular chloroplast genomes of *U. guianensis* and *U. tomentosa* showed the classical quadripartite structure with a large single copy (LSC) and a small single copy (SSC) separated by two inverted repeat regions (IRa and IRb) (Fig. 1 Supplementary File 1, Table S1 a). The genomic size of *U. guianensis* was 155,505 bp with an LSC of 86,043 bp and an SSC of 18,026 bp. The genome of *U. tomentosa* showed 156,390 bp with an LSC of 86,828 bp and an SSC of 18,126 bp. Both species showed IRs with 25,718 bp and GC content of 37.50%.

A total of 113 different genes were identified in the genomes of *U. guianensis* and *U. tomentosa*, with 131 genes in total when considering duplicates. Of this total, 84 are protein coding genes, 8 are ribosomal RNAs and 37 *tRNAs* (Fig. 1, Supplementary File 1, Table S1 b). The genes present in the chloroplast genomes of *U. tomentosa* and *U. guianensis* were categorized according to their function and the majority is involved in the process of replication and photosynthesis (Supplementary File 1, Table S2).

For the two species sequenced in this work, we found 18 genes with introns. The genes *clpP1* and *ycf3* were the only ones that showed two introns and three exons. The largest intron identified was on gene *trnK-UUU*, which encompasses the *matK* gene, with 2489 bp in *U. guianensis* and 2483 bp in *U. tomentosa.* The smallest intron was identified on gene *trnL-UAA* with 496 bp in both species (Supplementary File 1, Table S3). The only gene with intron present on the SSC was *ndhA*, while 12 other genes with introns were found on the LSC and 5 on the IRs.

### Comparative analysis of chloroplast genomes of the Cinchonoideae subfamily

In general, the size of the structures that make up the chloroplast genomes show few variations among the species of Cinchonoideae The smallest genome was of *U. rhynchophylla* with 154,605 bp^40^ and the biggest of *U. tomentosa* with 156,390 bp. *Mitragyna speciosa* showed the cpDNA with the biggest coding portion of 80,416 bp, while *Antirhea chinensis* showed the smallest with 79,980 bp. The non-coding portion varied from 74,211 bp in *U. rhynchophyll*a to 76,252 bp in *U. tomentosa* (Fig. 1a). Within all species of this subfamily, the IR’s sequence show the biggest portion of its content as non-coding regions whereas the LSC and SSC show coding regions as the biggest portion of its content (Fig. 1a). The GC content showed few variations among species. The total number of genes, ribosomal and transporter genes were the same in all seven species analyzed (Supplementary File 1, Table S1 b).

**Figure 2:**
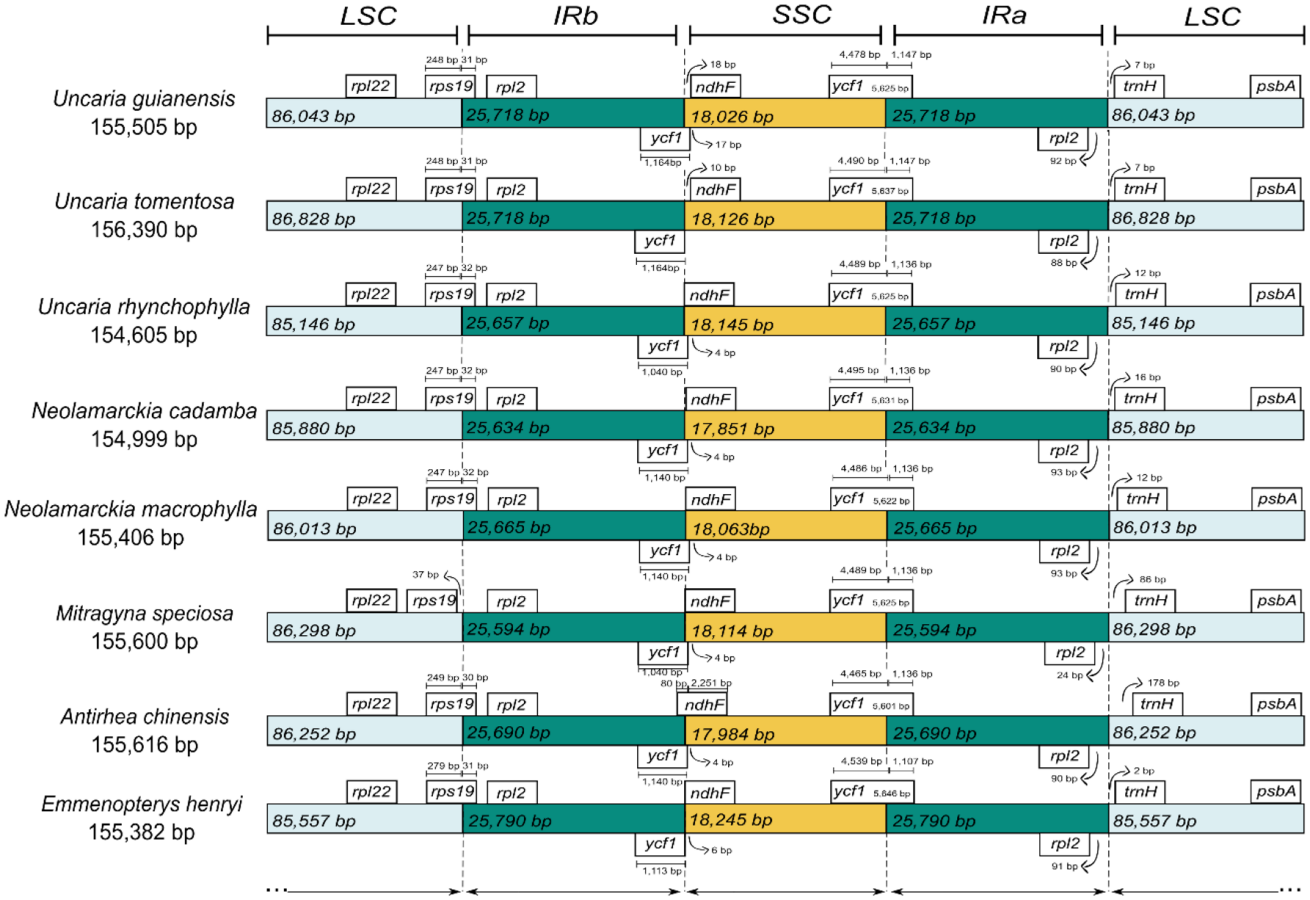
Comparison of the junction sites in the chloroplast genomes of seven species from the subfamily Cinchonoideae and one species (*Emmenopterys henryi*) from the sister subfamily Ixoroideae. The light blue bars represent the Large Single Copy (LSC), water green bars the Inverted Repeats (IRa / IRb) and yellow bars the Small Single Copy (SSC). The bars located at the top and at the bottom represent the genes transcribed clockwise and counterclockwise, respectively. The arrows indicate the distance, in base pairs, that the gene has surpassed or is distant from the junction sites.

The IR junction sites were compared among the seven species of Cinchonoideae, along with *Emmenopterys henryi* from Ixoroideae. The eight species showed the same genes within these junction regions. On the JLB junction (LSC / IRb), the *rps19* gene was present in the LSC and in a small portion of the IRb on most off the species, except for *Mitragyna spreciosa*, whereas the *rps19* gene did not invade IRb. On the JSB junction (IRb / SSC), the *ycf1* gene extended to the SSC on most of the species, except for *U. tomentosa*. For both *U. guianensis* and *U. tomentosa, ycf1* showed the biggest fragment in base pairs among all species (Fig. 2). The gene *ndhF* was inserted exclusively in the SSC in seven species but in *A. chinensis,* where this gene extended to the IRb. On the JSA junction (SSC / IRa) all species presented the gene *ycf1*. Lastly, on the JLA junction (IRa / LSC) of all species, the gene *trnH* was present and had a distance variation from the junction site from 7 to 178 bp (Fig. 2).

The alignment of the chloroplast genomes as locally colinear blocks (LCBs) performed in MAUVE (Supplementary File 2, Figure S1) showed collinearity among the genomes with conserved gene organization and content, without rearrangement among the species of the subfamily.

We found 44 high complexity repeats on the genome of *A. chinensis*, 28 on *Neolamarckia* sp., 29 on *U. guianensis* and *U. rhynchophylla*, 27 on *U. tomentosa* and 25 on *M. speciosa*. This indicates that *A. chinensis* is more divergent regarding the number of repetitions. For most species, the most frequent repeat was the forward one, followed by palindromic and reverse repeats. An exception is *M. speciosa* that showed palindromic repeats as the most frequent, followed by forward and reverse (Supplementary File 2, Figure S2 a). We could not identify any complemented repeat in *A. chinensis* and *M. speciosa*, while just one repeat of this kind was identified for the other species.

The repeat size varied from 30 to 912 bp in Cinchonoideae and it was possible to identify high complexity repeats in all regions of the genomes, except for *U. rhynchophylla* that did not show this type of repeat at the IRb. The region with the highest frequency of repeats was the LSC (Supplementary File 1, Table S4). *U. tomentosa* was the only species with repeat size bigger than 100 bp: a palindromic repeat of 119 bp and two forward repeats with 850 and 912 bp, all located in the LSC (Supplementary File 1, Table S4).

The total number of microsatellites repeats identified varied among genomes, with *A. chinensis* showing 33 elements and *U. rhynchophylla* 73. The size of these repeats varied from 10 to 27 bp (Supplementary File 1, Table S5). We could not identify hexanucleotides in any of the species, with mononucleotides being the most frequent type of motif within all species, followed by dinucleotide. The exceptions were *A. chinensis* and *M. speciosa* that showed tetranucleotide as the second most frequent type of motif (Supplementary File 2, Figure S2 b).

### Molecular evolution of the chloroplast genomes of the Cinchonoideae subfamily

**Figure 3:**
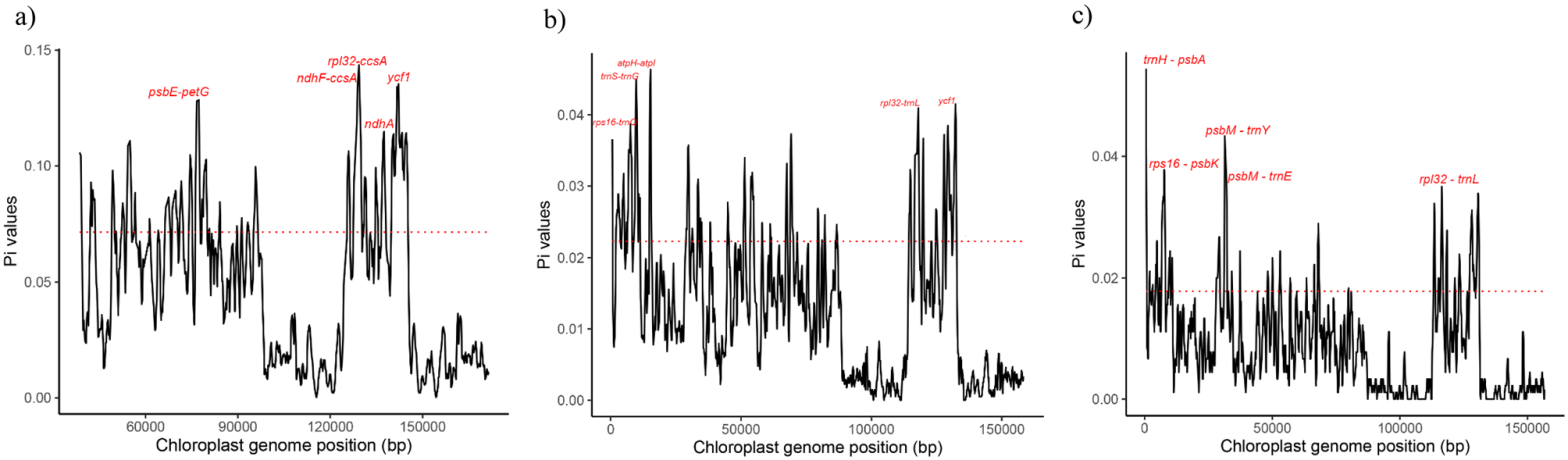
Mutational hotspots identified in the chloroplast genomes of the Rubiaceae family (**a**), of the Cinchonoideae subfamily (**b**), and of the *Uncaria* genus (**c**). The five regions with highest *π* values were plotted considering 2 × the median value as the cutoff point indicated by the red dashed line.

The mean estimated value for nucleotide diversity, that is, the divergency of the nucleotide composition within the chloroplast genomes of the family was *π* = 0.093. We identified 54 mutational hotspots (Supplementary File 1, Table S6). The regions with the highest values of *π* were *rpl32-ccsA* (*π* = 0.143), *ycf1* (*π* = 0.135), *ndhF-ccsA* (*π* = 0.135), *psbE-petG (π* = 0.128) and *ndhA* (*π* = 0.114) (Fig. 3a). For Cinchonoideae, the mean value was π = 0.030. We were able to identify 50 mutational hotspots within the subfamily (Supplementary File 1, Table S7) and the regions with the highest values of *π* were the four intergenic regions *atpH-atpI* (*π* = 0.046), *trnS-trnG* (*π* = 0.045), *rpl32-trnL* (*π* = 0.041), *rps16-trnQ* (*π* = 0.039) and the gene *ycf1* (*π* = 0.042) (Fig. 3b).

For the three species of *Uncaria*, the mean nucleotide diversity of the 74 mutational hotspots identified was *π* = 0.021, with the five regions with the highest values of *π* being all intergenic regions *trnH-psbA* (*π* = 0.054), *psbM-trnY* (*π* = 0.043), *rps16-psbK* (*π* = 0.038), *psbM-trnE* (*π* = 0.037) and *rpl32-trnL* (*π* = 0.035) (Supplementary File 1, Table S8; Fig. 3c).

**Figure 4:**
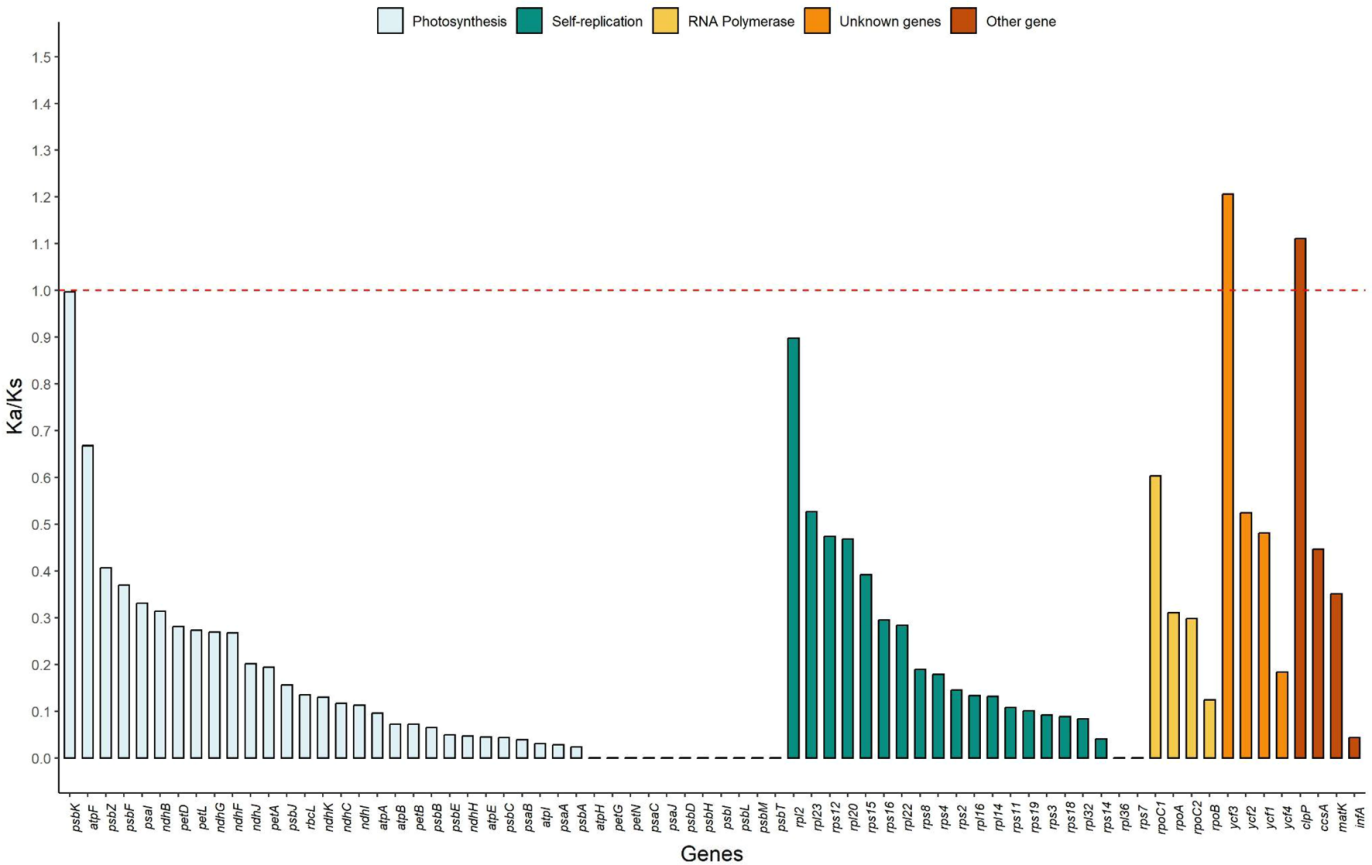
Ka/Ks ratio for seventy-two genes encoding plastid proteins shared by the species of the Cinchonoideae subfamily. The genes are separated by color of functional groups.

**Figure 5:**
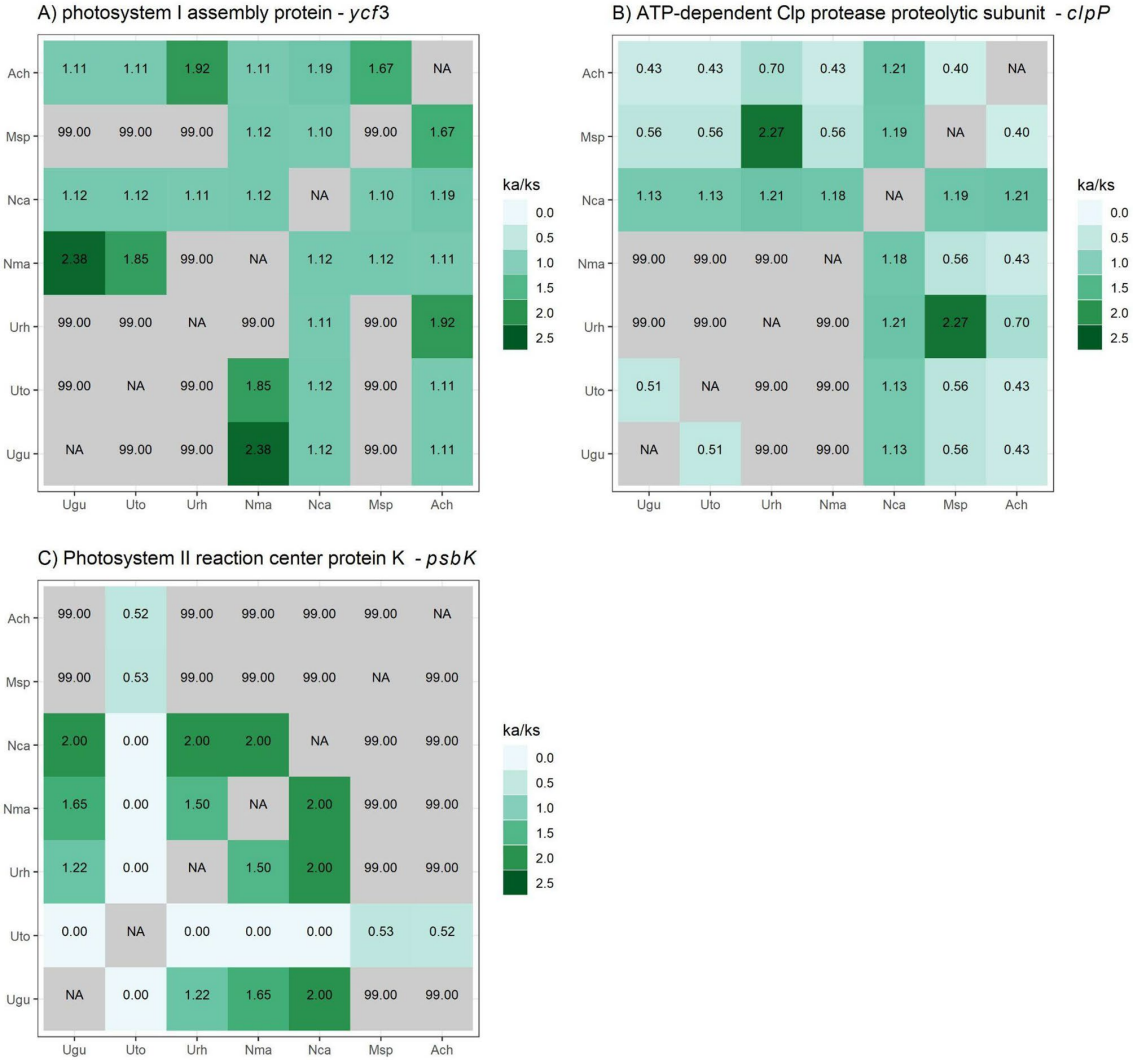
Ka/Ks ratio pairwise in chloroplast genes from seven Cinchonoideae (Rubiaceae). (**A**) Heat map Ka/Ks pairwise ratio in gene under positive selection *ycf3*. (**B**) Heat map Ka/Ks pairwise ratio in gene under positive selection *clpP*. (**C**) Heat map Ka/Ks pairwise ratio in gene under neutrally selection *psbK*. The value 99 represents the outliers with Ks values lower than necessary for the calculation of Ka/Ks. Ugu: *Uncaria guianensis*; Uto: *Uncaria tomentosa*; Urh: *Uncaria rhynchophylla*; Nma: *Neolamarckia macrophylla* ; Nca: *Neolamarckia cadamba*; Msp: *Mitragyna speciosa*; Ach: *Antirhea chinensis*;

For the genes shared by the seven species of Cinchonoideae, we estimated the mean rate of synonyms (*Ks* = 0.1158) and non-synonyms substitutions (*Ka* = 0.0346). *Ks* was more frequent than *Ka*, as already expected for chloroplast genomes. The mean non-synonyms and synonyms substitution rate (*Ka/Ks*) used phylogenetic information for all 72 protein coding genes shared by the species in this study varied from 0.0001 to 1.2061 (Fig. 4). The genes *ycf3* and *clpP1* are identified under positive selection, with *Ka/Ks* values of 1.2061 and 1.1109, respectively. The only gene closer to neutral selection (*Ka/Ks* = 0.9968) was *psbK*. Most of the chloroplast genes (69) from Cinchonoideae were found under negative selection (*Ka/Ks* < 1). (Supplementary File 1, Table S9). For the three genes under positive and neutral selection, we plotted heat maps in order to observe the pairwise relationships of Ka/Ks values (Fig. 5).

**Figure 6:**
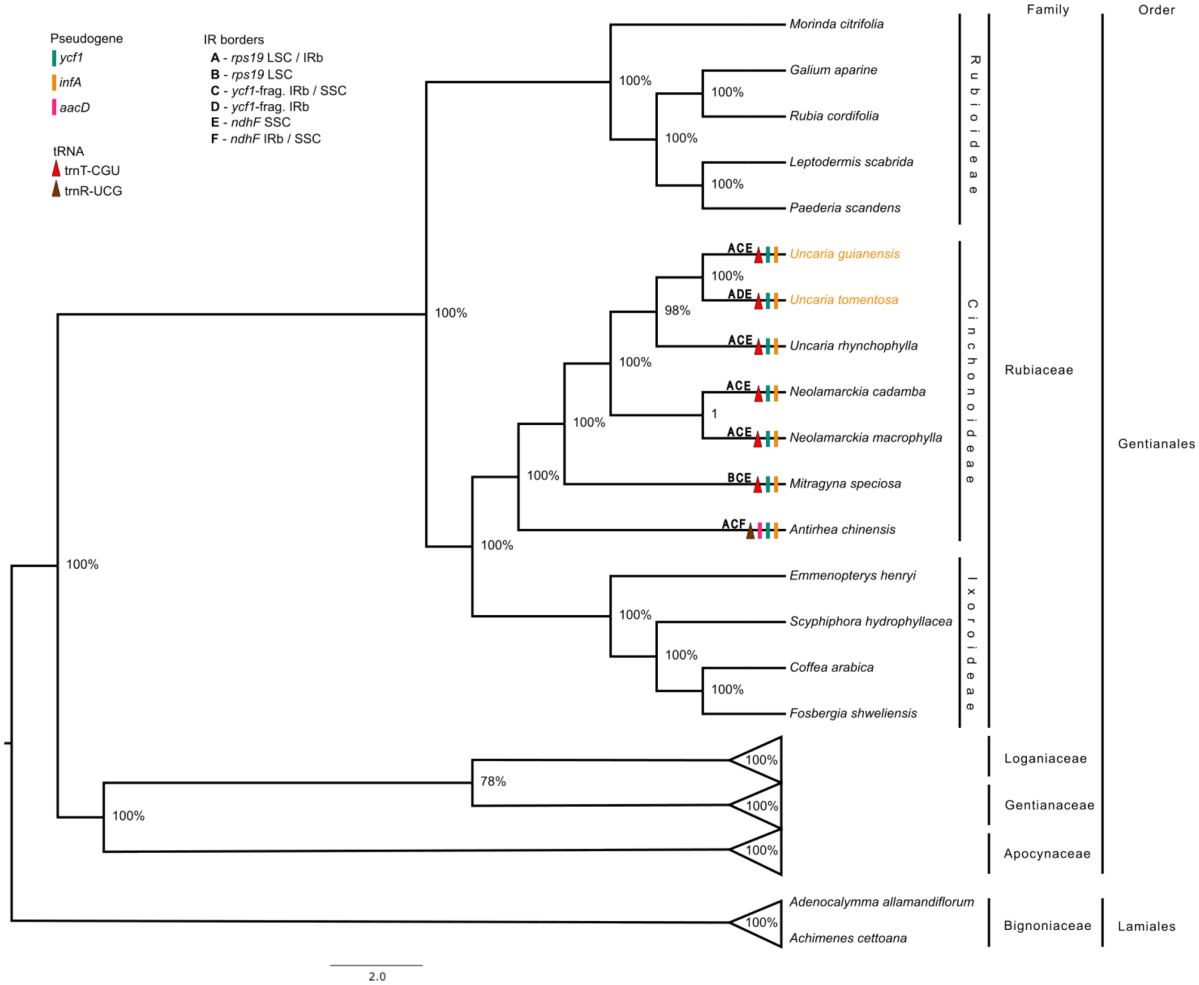
Reconstruction of the phylogenetic tree based on 78 protein encoding genes of the chloroplast genome of 44 species of the Gentianales order using the Maximum Likelihood method. *Adenocalymma allamandiflorum* and *Achimenes cettoana* are the species of the outgroup. The node numbers demonstrate the bootstrap support values. The triangles, rectangles and letters in the branches represent the differences in gene content and IR junction sites, plotted according to the subtitles in the figure.

The phylogenetic analysis performed by the method of maximum likelihood with the 78 genes recovered a congruent topology to APG IV. The order has five families but due to cpDNA sequences unavailability for Gelsemiaceae , we could not add it to the following analysis. The Gentianales phylogeny based on chloroplast genes showed an arrangement without polytomy among the sampled families and genera (Fig. 6). It is possible to notice two distinct clades within Gentianales, one composed by the Rubiaceae family and the other by the Loganiaceae, Gentianaceae and Apocinaceae families (Fig. 6). The Gentianaceae and Logoniaceae families form a monophyletic group. In general, we obtained high confidence values for most of the nodes, with bootstrap values varying from 78 to 100%. Within Rubiaceae, it was possible to obtain resolution for the three subfamilies. Both neotropical species, *U. guianensis* and *U. tomentosa* form a monophyletic group where *U. rhynchophylla* groups with them, since all these species belong to the same genus. *A. chinensis*, which is the most ancient species of all, showed a different *tRNA*, *trnR-UCG*, and an extra pseudogene, *accD*, when compared to the other species (Fig. 6).

### Primer validation for species of the Rubiaceae family

Considering the hypervariable regions within Rubiaceae (*ndhA, ndhF-ccsA, psbE-petG, rpl32-ccsA, ycf1, rps15-ycf1, ndhI-ndhA, rps4-trnL_UAA, trnT_GGU-psbD, petA-psbJ*) as targets and the parameters established for primer design, we could design ten primer pairs, one for each region (Supplementary File 1, Table S10). Of the ten primer pairs tested in silico, five (*ndhA*, *rpl32-ccsA, ndhI-ndhA, rps4-trnL* and *petA-psbJ*) were able to bind to more than 50% of the species used as templates (Supplementary File 1, Table S10). The pair for the region *ndhA* covered all the species (100%), while the primer pair for the *trnT_GGU-psbD* region covered the lowest number of species (6.2%). The primers for the regions *ndhF-ccsA* and *rps15-ycf1* did not bind, and therefore, were not considered able to cover any of the species. However, only one primer pair, *ndhA*, could bind to all the species used as templates within the analyzed subfamilies of Rubiaceae (Supplementary File 1, Table S11; Supplementary File 2, Figure S3).

When looking to the subfamilies individually, apart from *ndhA* that bound to all the species, the primers that best performed for Cinchonoideae were *rpl32-ccsA*, *rps4-trnL_UAA* and *petA-psbJ*, covering all the species analyzed, followed by *ndhI-ndhA*, that covered 83.33% of this subfamily (Supplementary File 1, Table S11; Supplementary File 2, Figure S3). For Ixoroideae, in addition to *ndhA*, *rpl32-ccsA* and *ycf1* also performed well by binding to all the species of this group (Supplementary File 1, Table S11; Supplementary File 2, Figure S3). For Rubioideae, *ndhA* was the only one binding to all of its representatives, followed by *ndI-ndhA* (Supplementary File 1, Table S11; Supplementary File 2, Figure S3).

None of the designed primers fulfilled all the physicochemical constraints analyzed here on this evaluation (Supplementary File 2, Figure S4). The primer for the *ndhA* region could bind to all the species but did not fulfill four physicochemical constraints. The primer pair *rps4-trnL_UAA* failed to fulfill the lowest number of constraints, however, it was able to bind only to 50% of the species, which were all the species of Cinchonoideae, one of Ixoroideae and none of Rubioideae (Supplementary File 1, Table S11; Supplementary File 2, Figure S3). The primer for the *ycf1* gene bound only to the species of Ixoroideae. Rubioideae was the group with less coverage by these designed primers. However, the three subfamilies had all its species covered by at least one primer pair.

## Discussion

In this work, we were able to build the chloroplast genomes of *Uncaria guianensis* and *Uncaria tomentosa* and to reannotate other chloroplast genomes available on NCBI for five other species of the Cinchonoideae subfamily. The gene content of *U. guianensis* and *U. tomentosa* was identical, as well as the number of introns within the genes. Thus, we can assure that the difference among their chloroplast genomes is in their intergenic regions. However, for other *Uncaria* species, it has been previously reported a variation in the number of genes, with *U. scandens* presenting the lowest number of genes (128) within the genus so far^41^. From the comparative analysis among the species of this subfamily, we found an evolutionary conservation of these genomes’ quadripartite structure: a LSC, a SSC and two IRs regions. Unlike what was found for the sister subfamily, Ixoroideae, where some tri-part cpDNA structures were found, showing a single IR^42^.

The analyzed species showed few variations on genome size and on the genes’ limits of the junction sites, supporting the concept that the dynamics of expansion and contraction of the IR regions at junction sites is the main factor driving the change on cpDNA size within angiosperms^43^. Closely related species tend to have similar genomic characteristics and the difference in the size of the LSC, SSC and IR regions among the species can only explain 20% of the variation on cpDNA size^44,45^. Within Cinchonoideae, *U. tomentosa* was the species with the longest cpDNA sequence, 156,390 bp, even when compared to other asian *Uncaria* species^41^, while *U. rhynchophylla* showed the shortest, 154,605 bp, but *U. scandens* has the shortest cpDNA for the genus so far (153,780 bp)41 . We found that the difference in genome size can be related to non-coding sequences. *U. tomentosa* and *U. rhynchophylla* showed the biggest and the smallest non-coding genome portion of the genome, respectively. Both species showed the same number of genes and introns which is still a greater number than most of the species of this subfamily. The size of the intergenic regions are the main factors responsible for the variation on cpDNA size in angiosperms^45^.

The presence or absence of introns within genes can help elucidate evolutionary processes of many species^46^. We identified 18 genes with introns in both species sequenced in this study, with *clpP1* and *ycf3* showing two introns. Two other species of the same subfamily, *N. cadamba* and *U. rhynchophylla,* also have 18 genes with introns^40,47^ while other species of *Uncaria* have *rps12* as an extra gene containing two introns^41^. In the species of the Ixoroideae subfamily, which also belongs to Rubiaceae, eight genes with introns were identified, two of which were the same as the ones identified in *U. guianensis* and *U. tomentosa*^42^. The *rps12* gene showed one intron and three exons, and exon 1 is located at the LSC and the others at the IRs. This gene went through a process called trans-splicing, which results on a gene structure found in most terrestrial plants^42,48–50^.

On the identification of high complexity sequences, we found that the species *A. chinensis* and *M. speciosa* did not show complement repeats, differing from its descendant's species. Overall, the size of the repeats was similar except for *U. tomentosa* that showed sequences of up to 912 bp, while for Chinese species of Uncaria, tandem repeats varied from 3 to 49 bp^41^. Most of the species of the sister subfamily, Ixoroideae showed variation on repeat size from 30 to 39 bp^42^. These sequences can be used in population and phylogenetic studies because they are important to look for the occurrence of rearrangements, a process that facilitates genomic recombination and results in chloroplast diversity^51,52^. Regarding low complexity repeats (SSRs), only *U. guianensis* and *U. Tomentosa* did not show pentanucleotide motifs, as well as it was previously seen for *U. scandens*, with only two repeats being the maximum found for this motif type in *U. rhynchophylla*. Unlike the Uncaria species analyzed here, tri- and tetranucleotide repeats were not found for some Chinese representatives of the genus ref. For all species, the most frequent motif type was mononucleotide, which is normally the most abundant repeat type in angiosperms chloroplast genomes^34,36,37,47,53^. Chloroplast microsatellites have been pointed out as highly informative regions, being useful for evolutionary, populational and genetic structure studies^54–56^.

The most basal species, *A. chinensis*, which belongs to the tribe Guettardeae, showed some divergences from other six species of the Naucleeae tribe: a different *tRNA*, the *trnR-UCG* and the *accD* pseudogene formation due to stop codons along the sequence. The gene *accD* encodes an acetyl-CoA carboxylase subunit and although some eudicots species have lost this gene^57^, it was shown to be essential for leaf development of tobacco (*Nicotiana tabacum*). In *Arabidopsis thaliana*, the lack of *accD* gene caused embryonic lethality, while in other species, the same was not seen due to the presence of a copy of this gene in its nuclear genome^58,59^. All species of the Cinchonoideae subfamily showed the pseudogenes *infA* and *ycf1* at the IRb/SSC. The *infA* encodes the protein responsible for the translation initiation factor 1 at the chloroplast and is an example of a chloroplast gene present at the nuclear genome^60^. The pseudogene *ycf1* is extended to the IRb/SSC and is commonly found at the cpDNA of other species too^47,61,62^.

The gene *ycf1* is considered a strong candidate for the molecular identification of land plants, acting as a barcode marker, and showing better performance than other markers such as *matK*, *rbcL* and *trnH-psbA*^24^, especially in phylogenetic relationship studies within a genus, such as *Pinus*^63^ and *Prunus*^64^. Our results show that *ycf1* is highly variable, however, this gene is located at the IR which compromises its effectiveness as a barcode. Besides, on our in-silico PCR analysis, the primer pair designed for this region only worked for one subfamily.

On our in-silico PCR evaluation step, the primers designed for the hypervariable regions of the Rubiaceae species showed that only one primer pair (*ndhA*) can cover all species within this family and is the best potential marker to be used on broader species identification studies of this family. However, other primer pairs showed reliable performance within each subfamily and our results present options of potential markers for cases of targeted research or limited financial resources. Still, caution is needed when using the other nine primer pairs designed here because to achieve complete taxonomic coverage using all of them, different regions need to be assessed and some species will still lack information of barcode sequences for some of them.

Using the cpDNA of sixteen species of the Rubiaceae family, we identified in this study the intergenic region *rpl32-ccsA* as having the highest value of nucleotide diversity. This region was also identified as hypervariable in other plant groups such as *Pterocarpus* (Fabaceae)^32^ and *Chaenomeles* (Rosaceae)^52^. Although it was the most hypervariable region, the primers designed for it were not efficient on covering all the species tested for Rubiaceae. The *ndhA* region, in which its primers covered all the Rubiaceae species, has already been used as a molecular marker and showed superiority when constructing phylogenetic trees than other widely used markers, such as *rbcL-accD*^65^. Besides, *ndhA* showed success as a species-specific marker for the identification of species of the *Curcuma* genus^66^, which encourages the usage of this region for the discrimination of species withinRubiaceae.

Using the cpDNA of three species of the genus *Uncaria*, we identified in this study, the intergenic region *trnH-psbA* as having the highest value of nucleotide diversity, as it was also seen for other representatives of the genus^41^ This region has already been tested through Blast and distance methods to be the more efficient on discriminating species of *Uncaria* than *rcbL, matK* and *ITS*^10^. The combination of *trnH-psbA* with *ITS2* also showed high applicability for authentication of plants from this genus, where four sequences of *U. tomentosa* were used on the dataset, along with one of *U. guianensis*^10^. Although *matK* and *rbcL* are the most recommended regions by the Consortium for the Barcode of Life^67^, it was not possible to determine a universal barcode for all plant species, since the identification effectiveness of these regions may vary among the taxonomic groups^25^. Thus, it is essential to test the hypervariable regions here identified for each target group by using coverage broad primers to confirm their potential to act as barcode markers, including the test on multiple individuals of each species.

For this dataset, the synonyms (*Ks*) substitutions were more frequent than the non-synonyms (*Ka*), that is, the nucleotide substitutions do not alter the produced amino acid, a result commonly observed in chloroplast genomes^34,37,68^. We identified two genes with positive selection, *ycf3* (1.2061) and *clpP1* (1.1109). The *ycf3* gene is related to an important gene in the process of the accumulation of the photosystem I (PSI) complex^69,70^. Moreover, the gene *clpP1* is related with plant development and active gene expression^69,71,72^. Although considering the phylogeny of *Uncaria* species and the *ycf3* and *clpP1* genes that are under positive selection, the pairwise relationships between the species of the genus *Uncaria* showed few mutations leading to the formation of outlier values. In addition, the gene *psbK*, shown to be under neutral selection, is related with photosynthesis, which usually show the lowest *Ka/Ks* ratio^69,73,74^.

Within Rubiaceae, it was possible to obtain the resolution of the three subfamilies as proposed by Bremer and Eriksson^1^ and as shown in the comparative analysis of the organellar genomes of Rubiaceae^40,41,47,75^.

## Conclusion

In this work, we performed the first assembly and characterization of the chloroplast genomes of the medicinal plant species *Uncaria guianensis* and *Uncaria tomentosa,* commonly known as cat's-claw. We also performed comparative analysis within the species of the Cinchonoideae subfamily. We concluded that the gene content and the chloroplast genome structure of the analyzed species are conserved and that most of the genes are under negative selection. Our in-silico PCR results show that to obtain complete coverage success within the Rubiaceae family, the primer pair for the *ndhA* region can be a promising tool. However, new tests should be made aiming at covering a greater number of species, along with the intra and interspecific discriminating power of each marker. The newly sequenced chloroplast DNA sequences make important genomic resources for evolutionary studies of the group.

## Material and methods

### DNA extraction and sequencing

*U. guianensis* and *U. tomentosa* seedlings in growth medium were given in by Reserva Particular do Patrimônio Natural (RPPN) Ecocerrado Brasil (Araxá – MG). Total genomic DNA extraction from the leaves was performed using CTAB 2% protocol^76^ and the extracted DNA was quantified by horizontal electrophoresis in a 1% Agarose gel and on Qubit® fluorometer 2.0 (Life Technologies). Genomic libraries were prepared using Nextera DNA Flex Library (Illumina) protocol and pair-end sequencing was performed using MiSeq® Reagent Kit v3 (600 ciclos) on a MiSeq Illumina platform at Laboratório de Genética e Biodiversidade – LGBIO, from Universidade Federal de Goiás, Goiânia—GO.

### Chloroplast genome assembly and annotation

The cpDNA was assembled using NOVOPlasty v.3.8.3^77^ with K-mer size of 39 using the *rbcL* sequence of each of the species as seed to begin extension and assembly (GenBank_uto: GQ852363, GenBank_ugu: AJ347007). The organellar genomes of *U. tomentosa* and *U. guianensis* were annotated with CHLOROBOX GeSeq^78^. Search settings included prediction of *tRNA* genes with tRNAscan-SE v.2.0.5^79,80^ and with ARAGORN v.1.2.38^81^, with the latter having maximum intron size set up as 3,000 bp and genetic code as “Bacterial / Plant plastid”. HMMER tool was used for predicting protein coding sequences (CDS) and ribosomal RNAs (rRNA), using as reference a chloroplast database of other embryophytes previously sequenced and annotated. Annotation results were manually checked using Geneious prime v. 2020.1.2 and pseudogenes classification was performed considering the presence of stop codons at sequence extension or partial gene loss. The circular cpDNA map was drawn using Organellar Genome DRAW (OGDRAW)^82^.

### Comparative analysis of Cinchonoideae subfamily

For the comparative analysis, chloroplast genomes of five species of Cinchonoideae already deposited on GenBank were used: *Uncaria rhynchophylla* (Miq.) Miq. ex Havil. (MN723865), *Neolamarckia cadamba* (Roxb.) Bosser (NC_041149.1), *Neolamarckia macrophylla* (Roxb.) Bosser (MN877388), *Mitragyna speciosa* (Korth.) Havil. (NC_034698.1) and *Antirhea chinensis (Champ. ex Benth.) Benth. & Hook.f. ex F.B.Forbes & Hemsl.*(NC_044102.1). The cpDNA of these species were annotated using the same parameters used for *U. guianensis* and *U. tomentosa* described in the previous topic and also had manually checks. MAUVE software was used to align the complete chloroplast genomes and to detect gene rearrangement.

The analysis of expansion and contraction of the inverted repeats regions (IRs) at junction sites was performed by comparing gene positions of the seven species onGeneious Prime^83^ and using as reference, the species of Ixoroideae, *Emmenopterys henryi*. To locate high complexity repetitive elements forward (F), reverse (R), palindromic (P) and complement (C), the software REPuter^84^ was used with a minimum repetition size of 30 bp and a hamming distance of 3 (90% identity). The microsatellite regions (SSR—simple sequence repeats) were predicted using MISA (MIcroSAtellite)^85^, setting up the minimum of 10 repeat units for mononucleotide, five repeat units for dinucleotide, four repeat units for trinucleotide and three repeat units for tetra-, penta- and hexanucleotide.

The cpDNA of all seven species of Cinchonoideae, including *U. guianensis* and *U. tomentosa* were aligned on MAFFT^86^ and then sent to DnaSP 6—*DNA Sequence Polymorphism*^87^ for searching hypervariable regions, using the following parameters: 600 bp as window length and 200 bp as step size. At the genus level, the identification of the hypervariable regions was performed using the same parameters with the alignment of three species of *Uncaria* (*U. guianensis, U. tomentosa* and *U. rhynchophylla*) and at the family level, with the alignment of the 16 species of Rubiaceae (Supplementary File 1, Table S12).

The threshold for the nucleotide diversity values (*π* – Pi) was determined as twice the median Pi values. The regions of high nucleotide diversity peaks were annotated with *Basic Local Alignment Search Tool* – Blast from NCBI. We estimated the *Ka/Ks* ratio to detect signal of selection in the common genes of the seven species of Cinchonoideae. The 72 protein-coding genes shared by the seven species were extracted and aligned separately on MAFFT^86^, and each alignment file of each gene was sent to PAML 4.9^87^ to estimate the *Ka/Ks* substitution rate of each one of them. We estimated the *Ka/Ks* ratio with neutral mode with tree information (runmode = 0, model = 0 and NSsites = 0) and with neutral mode without tree information (runmode = 0, model = 0 and NSsites = 0). Furthermore, we calculated the pairwise *Ka/Ks* for the 72 protein-coding genes and plotted heatmaps to better understand their relationship.

### Primer development and validation for the Rubiaceae family

We used the consensus cpDNA sequence alignment from 16 species of Rubiaceae and the hypervariable regions previously identified to design primer pairs. The primers were designed using the software *Primer3*^89^ bound to *Geneious Prime* v.2020.1.2^83^. The parameters defined for primer design were amplicon size of 350 to 600 bp, GC content of 30 to 60%, primer length of 20 to 25 bp and melting temperature of 52 to 62 °C.

Primers were evaluated for their range of taxonomic coverage and physicochemical properties on an in-silico PCR using the package openPrimeR in R^90^. The complete cpDNA sequence of sixteen species ofRubiaceae were used for this evaluation step, comprising three subfamilies (Rubioideae, Cinchonoideae and Ixoroideae). Each subfamily was considered a group and their species genome sequences were considered template sequences for the group.

The forward and reverse primers were evaluated in pairs. The allowed binding region for evaluating the primers was the program's default first and last 30 bp for forward and reverse primers, respectively. However, evaluation out of this target binding region was allowed so the whole genome was screened for binding events. The maximum number of allowed mismatches between a template sequence and a primer was five base pairs. Mismatches were forbidden on the last six base pairs of the primer's 3' end. For a group to be considered covered by a primer, we established the minimum of at least three species sequence bound to it, which represents 75% of the species analyzed for the smallest group (Ixoroideae). For the remaining primer settings and PCR conditions, we used the program's default settings.

### Phylogenetic analysis

We retrieved from the NCBI database 78 protein coding genes of the cpDNA of the order Gentianales to infer phylogenetic relationships within this group. We used 40 species of Gentianales (Supplementary File 1, Table S12) comprehending the Rubiaceae, Apocynaceae, Loganiaceae and Gentianaceae families. Two species of the Bignoniaceae family (order Lamiales) were used as outgroupsp. Overall, 44 species were used, including *U. guianensis* and *U. tomentosa*. The genes shared by the species were separated using Geneious^83^, aligned using MAFFT^86^ and concatenated on software Sequence Matrix^91^ for building a single matrix.

Informative sites were extracted on GBlocks v 0.91b^92^ and used as input on the software *Molecular Evolutionary Genetics Analysis* – MEGA version X (MEGA-X)^93^. The construction of the phylogenetic tree was performed on MEGA-X with 1,000 bootstraps. We chose the statistical method of maximum likelihood and the Tamura-Nei substitution model, assuming a uniform rate between sites. The consensus tree was visualized on FigTree^94^. The phylogenetic tree was used to plot divergent characters of cpDNAs among the species ofCinchonoideae, such as *tRNA*, pseudogenes and junction sites genes of the IRs.

## Supplementary Information

Supplementary Tables.

Supplementary Figures.

## Data Availability

The datasets generated and analyzed during the current study are available in the Genome Database on National Center for Biotechnology Information (NCBI) repository under the accession number OP794339 for *Uncaria guianensis* and OP794340 for *Uncaria tomentosa*. The BioProject and BioSample accession numbers on NCBI for *Uncaria guianensis* are PRJNA897943 and SAMN31594819, and for *Uncaria tomentosa* are PRJNA898287 and SAMN31605096, respectively.
