# Supplementary material for: Chloroplast genome characterization of Uncaria guianensis and Uncaria tomentosa and evolutive dynamics of the Cinchonoideae subfamily
Source: Sci Rep. 2023 May 24;13:8390. doi: 10.1038/s41598-023-34334-1 (PMC10209157; doi:10.1038/s41598-023-34334-1)
Supplement: Supplementary file 2 — Supplementary Figures. [file 41598_2023_34334_MOESM2_ESM.docx]

**Chloroplast genome characterization of *Uncaria guianensis* and *Uncaria tomentosa* and evolutive dynamics of the Cinchonoideae subfamily**

Andrezza Arantes Castro^2^, Rhewter Nunes^2,*^, Larissa Resende Carvalho^2^, Cíntia Pelegrineti Targueta de Azevedo Brito^2^, Ramilla dos Santos Braga-Ferreira^2^, Amanda Alves de Melo-Ximenes^2^, Leonardo Carlos Jerônimo Corvalán^1^, Bianca Waleria Bertoni^3^, Ana Maria Soares Pereira^3^, Mariana Pires de Campos Telles^2,4^

***** correspondence author

**Supplementary File 2, Figure S1** Additional figure

**Supplementary File 2, Figure S2** Additional figure

**Supplementary File 2, Figure S3** Additional figure

**Supplementary File 2, Figure S4** Additional figure


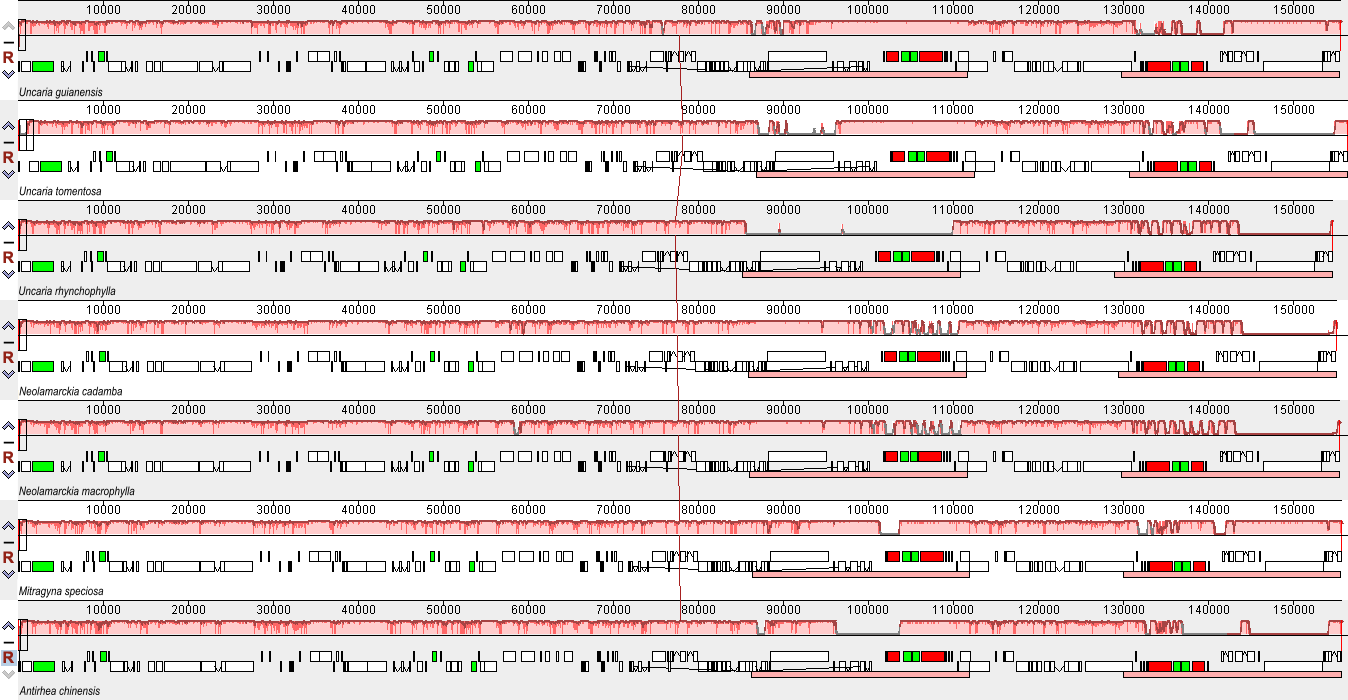


**Figure S1**. Alignment performed in the Mauve program of the chloroplast genomes of seven species of the Cinchonoideae subfamily. The locally collinear block (LCB) identified by the pink upper bars demonstrates the conservation of the segment, free from gene rearrangement and the red line shows the homology of the region. The green and red bars correspond to the transporter and ribosomal RNA genes, respectively.


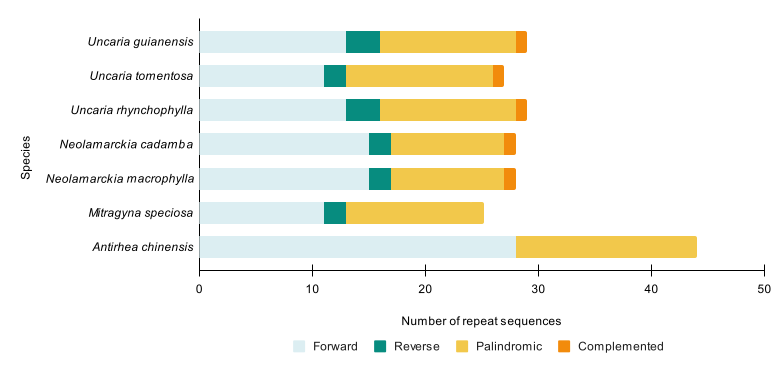


a)


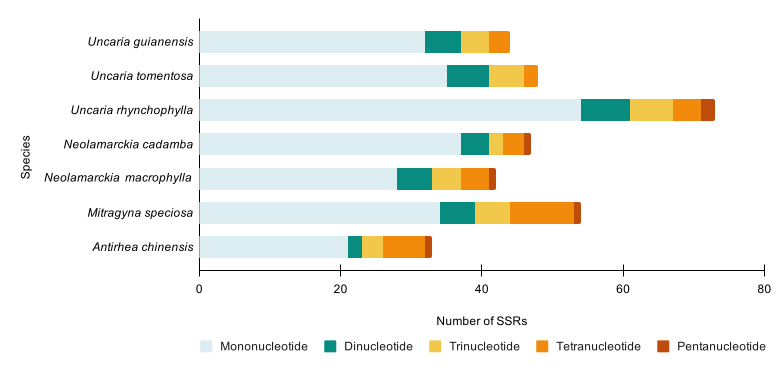


b)

**Figure S2.** Frequency of highly complex repetitions of forward, reverse, palindromic and complemented type (a) an microsatellites (b) within the species of the Cinchonoideae subfamily

**
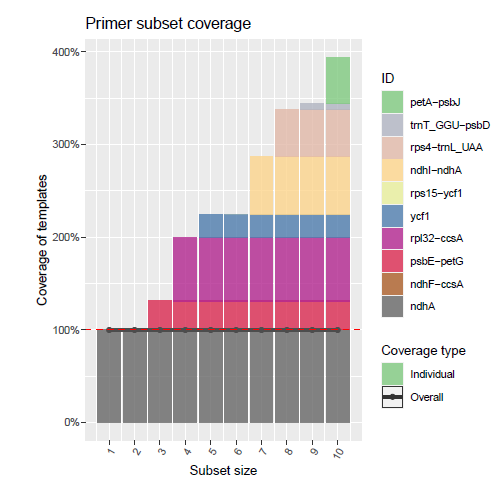
**

**Figure S3.** The coverage of optimal subsets of the input primer set. Here *optimal* refers to the fact that the subsets were selected such as to maximize the coverage. The line plot indicates the total percentage of covered template sequences, while the bars indicate he percentage of covered templates for individual primers. The cumulative coverage of the bars can exceed 100 percent because different primers may cover the same template redundantly. The target coverage ratio is indicated by the dashed horizontal line.


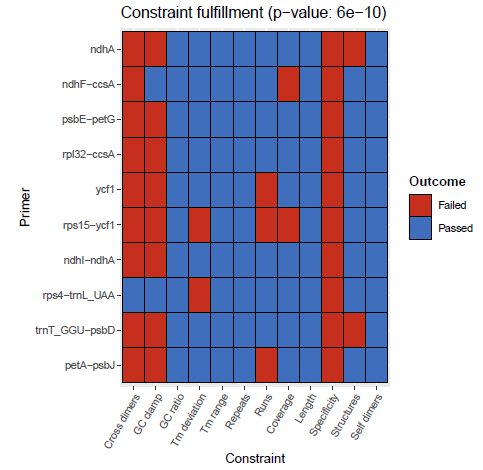


**Figure S4.** Restriction of physico-chemical properties for each of the tested primers. In red are constraints that the primers failed and in blue are constraints met.The provided p-value is an indicator of the overall quality of the primer set: significant p-values indicate primer sets fulfilling more constraints than reference primer sets from the literature.
